# Agreement among deep nasopharyngeal sampling culture results for 3 different swab types in preweaning dairy calves

**DOI:** 10.3168/jdsc.2023-0425

**Published:** 2023-10-16

**Authors:** Alejandro Hoyos-Jaramillo, Adriana Garzon, Heather M. Fritz, Barbara A. Byrne, Craig C. Miramontes, Terry W. Lehenbauer, Sharif Aly, Richard V. Pereira

**Affiliations:** 1 Department of Population Health and Reproduction, School of Veterinary Medicine, University of California–Davis, Davis, CA 95616; 2 California Animal Health and Food Safety Laboratory, University of California–Davis, Davis, CA 95616; 3 Department of Pathology, Microbiology and Immunology, School of Veterinary Medicine, University of California–Davis, Davis, CA 95616; 4 Department of Population Health and Reproduction, School of Veterinary Medicine, University of California–Davis, Tulare, CA 95616

## Abstract

Accurate isolation and identification of pathogens for an animal with bovine respiratory disease are of critical importance to direct appropriate decision-making related to the treatment of individual animals, as well as control and prevention options in a herd setting. The objective of this study was to compare nasopharyngeal sampling approaches to evaluate accuracy and agreement for the recovery of *Mannheimia haemolytica* (MH) and *Pasteurella multocida* (PM) from deep nasopharyngeal swabs (DNS) using 3 different swabs. Deep nasopharyngeal samples were collected from 45 dairy calves using 3 swabs: (1) double-guarded culture swab (DGS); (2) single-guarded culture swab (SGS); and (3) unguarded culture swab (UGS). To evaluate the degree of agreement between DGS, SGS, and UGS, culture results were compared for each calf sampled by using a kappa agreement test. Overall, findings from our study support that when using either SGS or DGS for DNS sampling of preweaning calves, a high agreement for recovery of PM is observed. A low recovery of MH was observed in the study, limiting the conclusion comparing the 3 DNS methods. Use of UGS is considered a potential alternative; however, a higher percentage of polymicrobial growth was found with UGS samples.

## Highlights

• Using single- or double-guarded swabs for deep nasopharyngeal sampling of preweaning calves shows a high agreement for recovery for *Pasteurella multocida* as evaluated by traditional culture methods.
• Unguarded swabs had a higher percentage of polymicrobial growth compared with guarded swab methods, and their use should take this finding into account.

## Graphical Abstract

**Figure:**
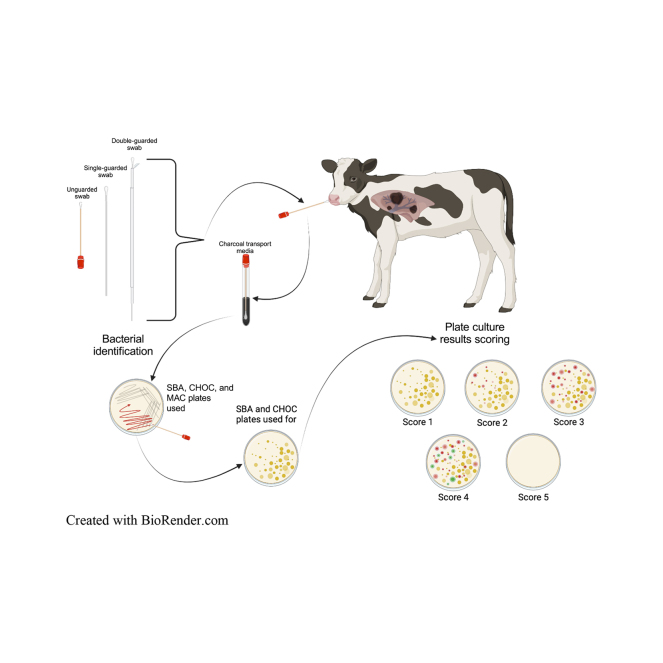
**Summary:** This article compares 3 different types of swabs for deep nasopharyngeal sampling of preweaning dairy calves. Findings from our study support that when using either single- or double-guarded swabs, there is a high agreement for recovery of *Pasteurella multocida*. Unguarded swabs are a potential alternative but result in a higher percentage of polymicrobial growth when cultured.

Bovine respiratory disease (**BRD**) is a multifactorial disease and can cause persistent negative economic impacts on the cattle industry due to higher morbidity and mortality rates of dairy calves and cattle in feedlots in the United States (Miles, 2009; Pereira et al., 2014; Peel, 2020). This multiagent complex is responsible for inducing clinical disease and lung pathology in young calves (Gershwin et al., 2015), leading to a decrease in feed intake and growth, increased antibiotic use and resistance, and higher mortality rates (Taylor et al., 2010; Pereira et al., 2020). Bovine respiratory disease is a complex disease involving the interaction between environmental factors, host immunity, and microbial pathogens (Taylor et al., 2010). *Mannheimia haemolytica* (**MH**) and *Pasteurella multocida* (**PM**) are the primary bacterial pathogens involved in BRD in cattle (Miles, 2009), and in preweaning dairy calves, PM is more frequently isolated compared with MH (Deepak et al., 2021). Furthermore, aerobic culture is a key diagnostic component to recover MH and PM, enabling further characterization such as in vitro antimicrobial susceptibility testing and genetic analysis (Loy et al., 2018). One of the most common techniques for BRD diagnosis is the collection of deep nasopharyngeal swab (**DNS**) samples by using long double-guarded or single-guarded swabs (Doyle et al., 2017). The advantage of using these guarded swabs is to reduce contamination from nostrils (double-guarded swabs) and to improve the accurate isolation of respiratory bacteria from DNS samples (Doyle et al., 2017).

The objective of this study was to compare nasopharyngeal sampling approaches to evaluate accuracy and agreement for the recovery of MH and PM from DNS using 3 different swabs. Deep nasopharyngeal samples were collected from a convenience sample of 45 dairy calves using 3 swabs: (1) double-guarded culture swab (**DGS**), (2) single-guarded culture swab (**SGS**), and (3) unguarded culture swab (**UGS**). To evaluate the degree of agreement between DGS, SGS, and UGS, culture results were compared for each calf sampled by using a kappa agreement test. Our hypothesis was that using SGS for DNS would generate high agreement for the recovery of MH and PM when compared with DGS, and that UGS DNS samples would have higher polymicrobial growth compared with either SGS or DGS.

The University of California Institutional Animal Care and Use Committee (IACUC; #22232) approved all experimental procedures conducted with animals for this study. A cross-sectional study design was used to collect DNS from one commercial dairy farm in California with a mixed herd, having Holstein, Jersey, and Jersey × Holstein crossbreds; this farm had a total milking herd of approximately 2,200 cows, with management practices commonly observed in California dairy farms. Calves enrolled in this study were housed in wooden hutches in a sequential fashion based on birth order, which allowed calves to have nose-to-nose contact with adjacent calves only.

Only female preweaning calves at or over 3 wk of age that did not have a farm history for treatment for any disease were included in the study. All calves were sampled at one of 4 distinct farm visits in May 2021. The sample size was based on the recovery of PM, and was based on an expected kappa of 0.8, with an expected standard error of 0.2, a prevalence of culture-positive for PM of 40%, and a confidence interval for significance at 95%, resulting in an estimated sample size of 37 animals.

At enrollment, calves were examined by a veterinarian (RP or AG) by conducting a visual and hands-on evaluation, including thoracic ultrasound (**TUS**) of the lungs. Visual examinations included using the University of California–Davis BRD scoring system for preweaning dairy calves when examining and scoring eye discharge, nasal discharge, ear droop or head tilt, cough, and respiratory effort (Aly et al., 2014; Love et al., 2014). Calves that were severely depressed to the point of being unable to stand or unresponsive, or comatose were not eligible to be enrolled in the study.

The TUS of the lungs was conducted by scanning the lungs using a portable ultrasound with a linear probe (Easi-Scan: Go, IMV Imaging Ltd.), as previously described (Buczinski et al., 2014; Ollivett and Buczinski, 2016). Briefly, a TUS score of 0 indicated a normally aerated lung, a TUS score of 1 indicated diffuse comet-tail artifacts without consolidation, a TUS score of 2 for findings with a consolidation ≥1 cm^2^ indicated lobular pneumonia, a TUS score of 3 for findings with 1 entire lung lobe consolidation indicated lobar pneumonia, a TUS score of 4 for findings with 2 entire lung lobe consolidation indicated lobar pneumonia and a TUS score of 5 for findings with ≥ 3 entire lung lobe consolidation indicated lobar pneumonia.

A convenience sample of 45 female calves was enrolled and sampled using all 3 swab types for deep nasopharyngeal sampling: (1) DGS (McCullough; Jorgensen Labs Inc.), (2) SGS (Guarded Culture Instrument, Kalayjian Industries Inc.), and (3) UGS. Two DNS were collected from one of the nostrils and one swab from the contralateral nostril.

The order and nostrils for which DNS swabs were collected were randomized by enrolling calves for sampling using a presampling generated list with random sampling order and nostril allocation, to reduce potential bias introduced by sampling order or number of times the same nostril was sampled. As an example, following allocation from the randomized list, a singular calf may have been sequentially sampled for a DNS on the right nostril using a DGS followed by a SGS, and with an UGS in the left nostril.

The DNS was collected using the selected swab by restraining the animal's head in a standing position. The animals' nostrils were wiped clean with a single-use paper towel and subsequently disinfected with 70% alcohol before inserting the sterile swab medioventrally in the nasal cavity until nasopharyngeal tissue was reached, and the swab was rotated several times against the mucosa. Swabs were immediately placed in Amies transport medium with charcoal (ACM, BD BBL CultureSwab Plus Transport Systems, Franklin Lakes, NJ), and transported in a cooler with ice to the laboratory for processing (Garzon et al., 2023).

Samples were submitted within 4 h to the CAHFS Laboratory in Davis, California, for aerobic culture, using standardized methods (Garzon et al., 2023). Each sample was cultured onto 5% sheep blood (**SBA**), chocolate agar (**CHOC**), and MacConkey agar (**MAC**). Plates were incubated for 24 h at 35 ± 2°C in 5% CO_2_. Organisms of interest included the respiratory pathogens MH and PM recovered from DNS (Remel, Thermo Scientific). For colonies that had a morphology consistent with PM or MH, the identity of colonies of the isolate was confirmed by biochemical testing and MALDI-TOF MS.

Plate culture results on the SBA and CHOC plates were used to score growth into one of 5 options: score 1, representing a pure growth with only one type of bacterial colony; score 2, with one dominant growth colony morphology, with other colony types present; score 3, with 2 dominant colony morphologies; score 4, with a polymicrobial growth, with >2 different dominant colony morphologies; and **NG**, representing no growth on the plates.

Statistical analyses were conducted using JMP 16.0 (SAS). Univariate linear regression models were used to evaluate the association of swab type (DGS, SGS, and UGS) and culture growth score (**CGS**). The odds ratio (**OR**) and 95% CI were calculated to determine the probability of association detection. For the calculation of the OR, a binomial variable was created for each of the 5 scores, that represented the presence (“1”) or not (“0”) of the referred CGS when comparing results between 2 different types of swabs. A value of *P* ≤ 0.05 was considered significant. A kappa statistic was conducted to evaluate the degree of agreement among DGS, SGS, and UGS for the recovery of MH and PM, respectively. A kappa agreement estimate was interpreted as poor if <0.40, fair to good if 0.41 to 0.75, or excellent agreement beyond chance if >0.75 (Flack, 1988; Aly et al., 2014).

Of the total of 45 calves, 36 had a BRD score of 0, 2 had a BRD score of 2, 6 had a BRD score of 4, and only one had a BRD score of 6, resulting in only one animal being defined as having BRD based on this scoring system. For TUS, only 2 of the 45 calves were diagnosed with BRD, having a TUS score of 2 and categorized as lobular pneumonia; both animals had abnormal lung sounds upon lung auscultation. No abnormal heart sounds were diagnosed upon auscultation. Using both the CA scoring system and TUS interpreted in parallel, a total of 3 animals were diagnosed with BRD in the study. Because of the low number of animals with BRD, the data were not stratified by BRD status.

The nasal passages of healthy calves contain many opportunistic bacteria including pathogens such as MH and PM (Griffin et al., 2010). Strains of these 2 bacteria are considered opportunistic pathogens for the development of BRD (Holman et al., 2015). Suppression of the host's immune system due to infections, changes in the environment, and management could potentially predispose to the rapid growth of these bacteria in the upper respiratory tract and be inhaled via droplets into the lungs where they may adhere to and colonize the epithelial surface, initiating pathogenesis (Griffin et al., 2010; Chai et al., 2022). Given the potential role of commensal pathogens for the development of BRD, a study by Schönecker et al. (2020) thought to evaluate the prevalence of antimicrobial resistance of opportunistic pathogens associated with bovine respiratory disease isolated from DNS of veal calves, given the risk they represent for development of BRD (Schönecker et al., 2020). Although our study did not focus on elucidating BRD pathogenesis but rather evaluate accuracy and agreement for the recovery for MH and PM, this information supports the relevance of using either calves with or without BRD for methodological comparison of DNS.

A significantly higher NG culture result was observed for SGS when compared with UGS (OR = 12.5; 95% CI: 1.5–102.9; Table 1). Although no other significant difference was observed for CGS among different swab types, UGS had a numerically higher percentage of samples with a score of 4, representing polymicrobial growth. Comparatively, a study by Van Driessche et al. (2017) observed that even when using bronchoalveolar lavage (**BAL**) for sampling the airway of calves, polymicrobial results could be observed (Van Driessche et al., 2017). Van Driessche's findings support that polymicrobial results are probably multifactorial, and not dependent only on type of DNS used. Having that in consideration, our results indicate that using UGS can be viewed as one of the factors that could increase the probability of polymicrobial growth when compared with SGS or DGS. A main concern of polymicrobial growth from a sample is that when present it can reduce the likelihood that the significant organisms causing disease will be effectively identified as the individual isolated colony of interest for further characterization, since other commensal organisms and contaminants may share similar colony morphology.

**Table 1:** The odds ratio for culture growth score comparison among swabs used for deep nasopharyngeal samples

Culture score^1^	UGS,^2^ % (n)	SGS,^3^ % (n)	DGS,^4^ % (n)		Type of DNS compared^5^	OR^6^	95% CI OR	*P*-value^7^
NG	2 (1)	22 (10)	13 (6)		DGS vs. SGS	0.54	0.18–1.6	0.27
					DGS vs. UGS	6.77	0.78–58.7	0.08
					SGS vs. UGS	12.57	1.5–102.9	0.02
1	11 (5)	20 (9)	27 (12)		DGS vs. SGS	1.45	0.54–3.9	0.46
					DGS vs. UGS	2.91	0.93–9.1	0.07
					SGS vs. UGS	2.00	0.61–6.5	0.25
2	24 (11)	22 (10)	20 (9)		DGS vs. SGS	0.88	0.32–2.4	0.80
					DGS vs. UGS	0.77	0.28–2.1	0.61
					SGS vs. UGS	0.88	0.33–2.3	0.80
3	36 (16)	22 (10)	29 (13)		DGS vs. SGS	1.42	0.55–3.7	0.47
					DGS vs. UGS	0.74	0.3–1.8	0.50
					SGS vs. UGS	0.52	0.2–1.3	0.17
4	27 (12)	14 (6)	11 (5)		DGS vs. SGS	0.81	0.23–2.9	0.75
					DGS vs. UGS	0.34	0.11–1.1	0.07
					SGS vs. UGS	0.42	0.14–1.2	0.12

1 NG represents no growth; score 1, representing a pure growth with only one type of bacterial colony; score 2, with one dominant growth colony morphology, with other colony types present in small numbers; score 3, with 2 dominant colony morphologies; score 4, with a polymicrobial growth, with >2 different dominant colony morphologies.

2 Culture growth score percent and count distribution for unguarded culture swab (UGS).

3 Culture growth score percent and count distribution for single-guarded culture swab (SGS).

4 Culture growth score percent and count distribution for double-guarded culture swabs (DGS).

5 Type of deep nasopharyngeal swab (DNS).

6 OR = odds ratio for the referred culture growth score. The first swab type served as the reference, so a number above 1 indicated a higher OR for samples collected using that swab type to have the culture growth score considered.

7 *P*-value for the OR.

For PM, recovery percentages were 40% (18/45), 44% (20/45), and 44% (20/45) for UGS, SGS, and DGS, respectively. Agreement for PM culture results among all 3 swab types (UGS, SGS, and DGS) was 51.1% for culture-negative and 37.8% for culture-positive (Table 2). High kappa agreement values (all above 0.8) were observed among the 3 methods tested, as presented in Table 3. Similar findings have been observed by Crosby et al. (2022) when comparing 1 double-guarded and 2 different unguarded swabs for DNS collected from beef-type steers 2 wk after feedlot arrival, with an observed complete concordance in culture results for the 3 sampling methods for 77% of cattle enrolled in the study (Crosby et al., 2022).

**Table 2:** Concordance and pattern results among swab types UGS, SGS, and DGS for *Pasteurella multocida* (PM) and *Mannheimia haemolytica* (MH) culture results for 45 preweaning calves sampled for deep nasopharyngeal swab (DNS)

Culture result^1^	PM^2^ culture, n (%)	MH^3^ culture, n (%)
NNN	23 (51.1)	42 (93.3)
YYY	17 (37.8)	—
YNY	1 (2.2)	—
NNY	1 (2.2)	2 (4.4)
NYN	2 (4.4)	—
YNY	1 (2.2)	1 (2.2)

1 Agreement among culture results (either culture-positive or -negative) in order for unguarded culture swab (UGS), single-guarded culture swab (SGS), and double-guarded culture swab (DGS), respectively. “N” indicates no growth, and “Y” indicates growth.

2 Culture frequency or percent for DNS swabs for *Pasteurella multocida*.

3 Culture frequency or percent for DNS swabs for *Mannheimia haemolytica*.

**Table 3:** Kappa results comparing 3 deep nasopharyngeal swab approaches for the culture of *Pasteurella multocida* (PM) and *Mannheimia haemolytica* (MH)

Groups compared (vs.)^1^		PM		MH	
		Kappa^2^	95% CI	Kappa^2^	95% CI
UGS	SGS	0.81	0.65–0.97	0	—
UGS	DGS	0.90	0.78–1.0	0	—
SGS	DGS	0.82	0.66–0.97	0.48	0.63–1.0

1 UGS = unguarded culture swab; SGS = single-guarded culture swab; DGS = double-guarded culture swab.

2 Kappa agreement.

For MH, a low recovery from samples collected was observed, with only 3/45 positive from DGS, 0/45 from SGS, and 1/45 from UGS. This finding is in agreement with a study by Schönecker et al. (2020), where MH was isolated from only 1.7% of DNS collected from healthy veal young calves with <91 d of age (Schönecker et al., 2020). Agreement for MH culture results among all 3 swab types (UGS, SGS, and DGS) was 93.3%, all being for culture-negative results (Table 2). This significantly limited the ability to evaluate the recovery agreement among the 3 methods evaluated for MH as seen in Table 2, Table 3 and is a study pitfall and a consequence of collecting samples opportunistically from mostly healthy animals. The one animal sampled with a culture-positive result from UGS matched with DGS, both with culture-positive results. Only 3/45 calves had a culture-positive result for both MH and PM, with 2/3 only being observed for DNS collected using DGS, and 1/3 for both DGS and UGS.

Overall, findings from our study indicate that when using either SGS or DGS for DNS sampling of preweaning calves, there is a high agreement for recovery of PM as evaluated by traditional culture methods. A potential concern when selecting a swab is that UGS could result in a higher probability for having polymicrobial growth. Additional factors that may be considered when selecting a swab type for DNS in livestock production settings include cost-effectiveness and ease of use in large herd populations.

An advantage of SGS over DGS includes an easier and quicker sample collection, given the lack of extra layers of protection, which increases handling of the swab while simultaneously restraining the animal, and the lower cost of SGS when compared with DGS (~$1 US less expensive per swab in our study).
